# Delayed First Milking in Unassisted Overnight Calving Did Not Affect the Quality of Colostrum but Influenced Serum Brix Refractometry in Holstein Calves at Two Days of Life

**DOI:** 10.3390/ani12131665

**Published:** 2022-06-28

**Authors:** Daniel Gustavo Manosalva, Luca Grispoldi, Marco Spagnolo, Martina Crociati

**Affiliations:** 1 Società Agricola Nonno Ciro, Località Foreste Vecchie, 61020 Montecalvo in Foglia, Italy; danimanosalva@hotmail.com (D.G.M.); marcospag@yahoo.it (M.S.); 2 Dipartimento di Medicina Veterinaria, Università degli Studi di Perugia, 06126 Perugia, Italy; grisluca@outlook.it; 3 Centro di Ricerca di Medicina Perinatale e della Riproduzione, Università degli Studi di Perugia, 06122 Perugia, Italy

**Keywords:** calving management, colostrum, calf, Brix refractometry, immune passive transfer

## Abstract

**Simple Summary:**

The study aimed to evaluate whether night-occurring calving and delayed first milking affected colostrum quality and immune passive transfer, measured through Brix refractometry. According to the Generalized Linear Model, parity ≥ 4, calving months of March, April, and from September to November positively influenced the quality of colostrum. Dams carrying a male calf produced lower quality colostrum compared with those carrying a female calf (−2.78 ± 1.04 Brix%, *p* = 0.008); heavier female calves were associated with greater Brix% of colostrum (0.29 ± 0.05 for each kg increase, *p* < 0.001). Night- or day-calving had no effect on the Brix% of colostrum. The only factor influencing the serum Brix% of female Holstein calves at two days of life was the day- or night-occurring birth (−0.386 ± 0.188 Brix% in calves born during the night, *p* = 0.04). Our results showed that calves born overnight and fed the day after had decreased serum Total Protein concentrations as indicated by reduced Brix refractometer readings, compared with calves born during the day. However, the administration of 4 L of high-quality colostrum proved to be effective in ensuring a serum Brix > 8.4% at two days of life.

**Abstract:**

Timely administration of good-quality colostrum represents the first farm strategy to avoid the failure of passive transfer (FPT). However, calves born during the night are likely to be fed later than recommended. Our aim was to evaluate whether night-occurring calving and delayed first milking affected colostrum quality and immune passive transfer. The dataset included 463 calvings. Four liters of colostrum were administered by an esophageal tube feeder. The mean Brix% of colostrum was 27.43%, while serum Brix% at two days of life in calves was 10.19%. According to the Generalized Linear Model, parity ≥ 4, calving months of March, April, and from September to November positively influenced the quality of colostrum. Dams carrying a male calf produced lower quality colostrum compared with those carrying a female calf (−2.78 ± 1.04 Brix%, *p* = 0.008); heavier female calves were associated with greater colostrum quality (0.29 ± 0.05 for each kg increase, *p* < 0.001). Night- or day-calving had no effect on the quality of colostrum. The only factor influencing the serum Brix% of female Holstein calves at two days of life was the day- or night-occurring birth (−0.386 ± 0.188 Brix% in calves born during the night, *p* = 0.04). Our results showed that calves born overnight and fed the day after had decreased serum Total Protein concentrations as indicated by reduced Brix refractometer readings, compared with calves born during the day and fed quickly after birth. However, the administration of 4 L of high-quality colostrum likely improved their serum Brix% at two days of life. Alternatively, where the prevalence of good-quality colostrum is lower, improving calving supervision and ensuring timely feeding are important to reduce the risk of FPT.

## 1. Introduction

Timely administration of good-quality colostrum represents the first and most important farm management strategy to ensure the calf is protected [1,2]. Calf ability to absorb immune-globulins (i.e., IgG) from colostrum progressively decreases, due to the advancing impermeability of intestinal barrier [3].

Radial immunodiffusion (RID) and turbidimetric immunoassay (TIA) represent the gold standard for IgG measurement [3,4,5], but these methods are expensive, difficult to implement routinely at the farm level, and both require more than 24 h for results to be obtained. Optical and digital refractometry can estimate the total solid content of a solution. Serum and colostrum can be assessed for Total Protein content and, in the last instance, for a proxy measurement of immune-globulin concentration [6]. Good reliability between RID and Brix refractometry has been demonstrated for the evaluation of colostrum quality and for calf serum IgG estimation [3,5]. For this reason, Brix and digital refractometry represent the most common approaches for the indirect evaluation of colostrum quality and calf serum I IgG estimation [7]. The cutoffs for colostrum and serum Brix refractometry are still under debate among scientists. However, a Brix > 21% identifies a good-quality colostrum (IgG ≥ 50 g/L), while Brix > 8.4% for calf serum (IgG ≥ 10–12 g/L) is representative of a good passive transfer within the first week of life [1,5,8]. The interval between calving and first milking can influence the IgG content of colostrum due to a progressive dilution effect, particularly when collection is carried out more than 6 h after parturition [6]. Milking dams as soon as possible after calving is recommended to ensure a good-quality colostrum. This goal can be easily reached when calving occurs during the day, due to visual observation of the maternity area by personnel. Calving occurring during night hours, approximately from 18:00 to 05:00, is usually unmonitored and colostrum collection and administration is delayed until the following morning, even 10–12 h after parturition [9,10].

Calves that receive colostrum later than at 6 h of life are at increased risk of failure of passive transfer (FPT) of immunity and of morbidity and mortality. Reducing calf mortality optimizes the management of replacement heifers, thus improving the genetic selection and the overall farm net return. Given the economic value of heifers, many farms focus on improving the management of newborn females, such as checking serum Brix% in the first week of life [3]. This study aimed to evaluate whether unmonitored night-occurring calving and delayed first milking had an effect on the following: (i) colostrum quality measured through Brix refractometry, and (ii) the transfer of passive immunity assessed through serum Brix refractometry in female Holstein calves at two days of life.

## 2. Materials and Methods

### 2.1. Animals and Husbandry

The study was conducted in a dairy farm located in the Marche Region, Italy (43°47’08.3” N, 12°38’22.4” E), during the period from 2020 to 2021. Ethic review and approval were not applicable according to the directive 2010/63/EU: the blood samples were collected from the farm veterinarian during routine health-check procedures, the study did not involve us housing animals, and the procedures on-farm involved routine, non-invasive practices. The herd size consisted of n = 270 lactating Holstein cows; the mean milk yield was 37.4 kg/head/day with 3.84% fat and 3.45% protein, 26,000 colony-forming units, and 169,000 somatic cell count. The mean age for the first pregnancy was 14.3 months; heifers were inseminated with Holstein bull sexed-sorted semen, while multiparous cows received Holstein or beef bull semen. 

Both lactating and dry cows were housed in free-stall barns with straw bedding; pregnant heifers and multiparous cows were kept separated in two dedicated pens. Replacing heifers were housed in free-stalls with straw bedding. Dams were moved to the dry area approximately 59.73 ± 16.19 days before the expected calving date; steaming-up extended from 20 days before to 20 days after calving both for primiparous and multiparous cows. First parity animals were kept separated from multiparous ones. From 35 to 45 days before the expected calving date, dams received vaccinations against *E. coli* K99, bovine Coronavirus, and Rotavirus (Bovilis Rotavec Corona, MSD Animal Health s.r.l., Milan, Italy). A total mixed ration (TMR) was distributed once per day and moved toward the feed bunk frequently by a robot.

The maternity area was monitored once per hour during the day. During working hours (05:00 to 18:00), each time a cow was recognized as being at the beginning of labor, i.e., showing vaginal discharge, laying down with abdominal contraction, or fetal sacs or fetus partly outside the vulva, visual monitoring of calving progress was performed every 30 min. If no progress was noticed after two consecutive checks, the parturient cow was obstetrically examined and the degree of dilation of the birth canal and the presentation, position, and posture of the fetus were assessed. Obstetric assistance was provided according to the recognized obstetrical procedure [11,12]. The degree of calving difficulty was scored as “eutocia”, “mild dystocia” (prolonged expulsive phase or fetal abnormal presentation), and “severe dystocia” (feto-maternal disproportion, uterine torsion, or cervical stenosis) [11,13].

For all calves, the sire breed and sex were recorded; in calves born during the day from monitored and assisted delivery, the hour (hh:mm) was also included in the dataset. Female Holstein calves were weighed (KERBL Digital scale, Albert Kerbl GmbH, Felizenzell 9, 84428 Buchbach, Germany). First neonatal care was provided, including the application of diluted iodine solution on the umbilical cord (7.5% povidone–iodine solution, Betadin Meda Pharma S.p.A., Milano, Italy). The calf was immediately separated from the dam and housed in an individual calf pen with abundant straw bedding. Fresh water was provided after birth. The dam was also checked for the integrity of the birth canal, then she was milked through a portable milking system. An aliquot (10 ml) of colostrum was collected before calf feeding, for Brix refractometer evaluation (Optical Refractometer Brix ATC, Agritech Store, Trento, Italy). Colostrum containing blood or clots indicative of udder inflammation [14,15] was excluded from the study, as colostrum from cows already suckled by their calf. Calves that fed directly from their dams were identified due to the presence of meconium [16] and/or due to a lack of teat sealant for dry cow therapy at any quarter (in multiparous cows); in such cases, data both from the dam and calf were excluded from further analysis. Four liters of colostrum were administered to the calf soon after collection by an esophageal tube feeder (Perfect Udder Deluxe Esophageal Feed Tubes, DLM s.r.l., Lodi, Italy), within 1 h of life. In case colostrum quality (Brix ≤ 21%) or quantity was considered unsatisfactory, 4L of frozen colostrum were thawed and administered as described above. Here, the Brix% of the thawed colostrum was included in the database.

In calves born overnight from unmonitored and unassisted parturition, these procedures were delayed to the following morning.

Female Holstein calves were submitted to venous blood collection at two days of life. Blood (10 ml) was collected by a jugular vein puncture through a sterile 16g needle connected to a Vacutainer empty tube (BD Vacutainer Systems, Plymouth, UK) and stored at 4 °C. Samples were centrifuged for 15 min at 3000× *g* within 1 h after collection; sera obtained were then used for Brix refractometry evaluation.

Calves were housed in individual pens until 30 days of life; boxes were provided with infrared lamps for the first 3 days of life during the cold season. At 30 days of life, female calves were moved in four-calf boxes until weaning (84.64 ± 3.54 days of age; 113.12 ± 9.84 kg; average daily gain: 0.912 ± 0.091 kg/day), then they were raised in multiple groups. All calves received trimming of the tail to improve hygiene.

### 2.2. Data Collection and Statistical Analysis

Data concerning the dam’s ID, parity, date of calving, hour (for day-calving (hh:mm); continuous variable), dry period length, twinning, calf sex and sire breed, and death and disease events were recorded. For purebred female Holstein calves, a subset was created including weight at birth and serum Brix evaluation at two days of life. The dataset was created using Office Excel™ Software (Office 2010, Microsoft Corporation, Redmond, WA, USA). The dataset was then analyzed through IBM® SPSS v23 Software. Night-calving events were assigned 00:00 h as the default. To better explore the effect of day- versus night-calving on the Brix evaluation of colostrum, all calving events were also assigned the dichotomous variable “DayNight”, that is, 1 = day (05:00–17:59) and 0 = night (18:00–04:59).

The distribution of parity, hour of calving, month of calving, Brix refractometry for colostrum and serum of calves at two days of life, and weight of female purebred Holstein calves were plotted and analyzed by the Explore function. Outliers, when identified, were excluded from further analysis.

Multivariable logistic regression was used to assess the association between: (i) calf sex, sire breed, dam parity, and the outcome DayNight; (ii) calf sex, sire breed, dam parity, and the occurrence of dystocia. The Generalized Linear Model (GENLIN procedure) was used to evaluate the associations between: (*i*) the Brix refractometry of colostrum (dependent variable), dam parity, month of calving, DayNight, and calf death (as fixed factors), and weight and dry period length (as covariates); (*ii*) the Brix refractometry of serum of calves at two days of life (dependent variable), dam parity and Brix refractometry of colostrum fed (as fixed factors), and dry period length of the dam and calf weight (as covariates).

The result of each test was considered statistically significant when *p* < 0.05.

## 3. Results

During the observation period, 476 calvings were observed, but 13 of them were not included due to calf suckling before colostrum collection. A total of 463 calving events were then entered into the database, with 161 of them belonging to primiparous cows (34.77%). Table 1 shows the characteristics of the distribution of dam parity, Brix refractometry of colostrum from each dam and fed to calves, Brix evaluation of calves’ serum at two days of life, and female Holstein calves’ weight. A total of 10 out of 463 calvings were twin births, for a total of n = 473 calves born and a twinning rate of 2.16%. A total of 29 out of 473 calves died in the first 30 days of life (6.1%). A total of 9 deaths were associated with twinning, n = 18 were due to pneumonia or diarrhea. One male calf was affected by severe congenital malformation and was euthanized. As shown in Table 2, 49 calvings (10.58%) were classified as “mild dystocia”, while 1 calving from a multiparous cow (parity: 5), which occurred at 23 days beyond the predicted term, was classified as “severe dystocia”. A female Belgian-blue crossbred calf was born after forced extraction and died at 12 h of life. A total of 10 out of 463 dams (2.16%) produced unsatisfactory colostrum from a qualitative perspective (Brix < 21%), while in 32 cases (6.91%) the amount of colostrum collected was less than 4 L.

**Figure 1:**
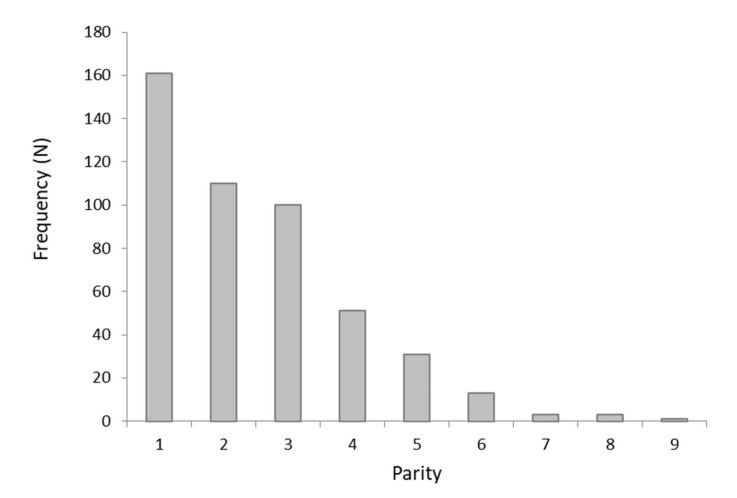
Distribution of parity.

Figure 1 shows the distribution of dam parity; primiparous cows accounted for 34% of the records (n = 161), while second and third parity cows were 23.3% (n = 110) and 21.1% (n = 100) of the herd, respectively.

**Figure 2:**
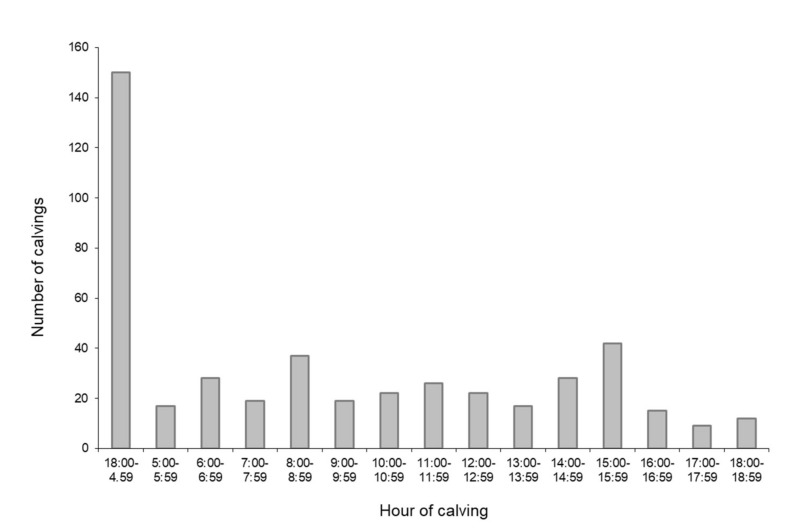
Distribution of calving hour during the day.

During the study, 313 calving events occurred during the day (67.6%) and 150 occurred at night (32.4%), as shown in Figure 2.

**Figure 3:**
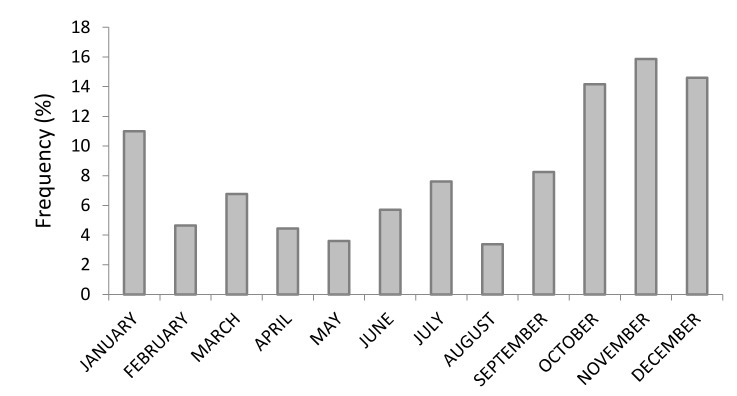
Distribution of calving events during the year.

Figure 3 shows the distribution of calving across the year. A greater percentage of calving occurred from October to January, while the opposite was observed in August.

A total of 296 female calves (62.6%) and 177 male calves (37.4%) were born. Among the female calves, 189 were Holstein purebred. Overall, 48.8% of the calves born were Holstein, 30.0% of the calves were Belgian-blue crossbred, with smaller percentages of Limousin (9.1%), Wagyu (7.4%), and Angus (3.0%) crossbreds.

**Figure 4:**
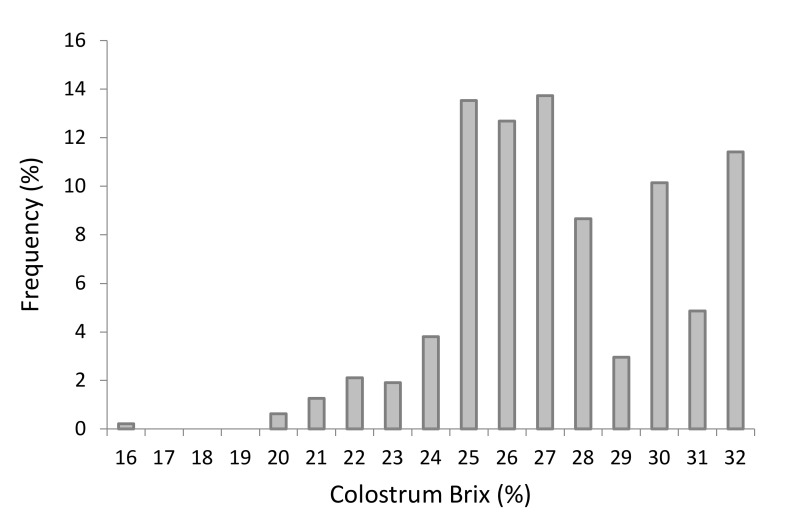
Distribution of Brix% of colostrum collected from dams.

The distribution of the Brix assessment of each dam’s colostrum, as reported in Figure 4, showed a right-shift, with most values ≥ 27% Brix.

**Figure 5:**
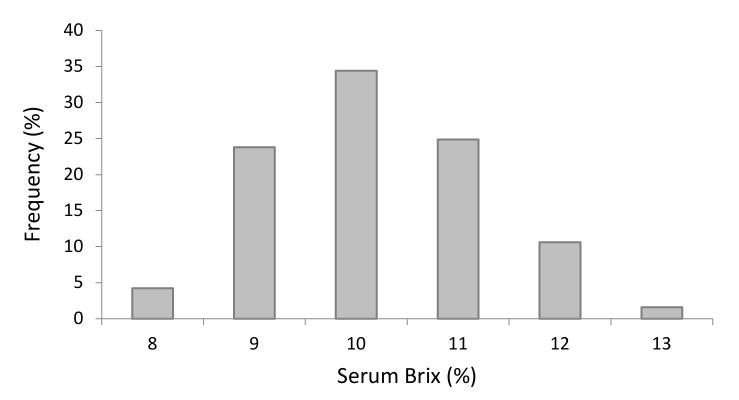
Distribution of Brix% of female Holstein calves’ sera.

The distribution of serum Brix refractometry of female Holsteins at two days of life is shown in Figure 5.

The results from the multivariable logistic regression are included in Table 3. Male calves showed slightly greater odds for being born during the daytime (OR: 1.473) compared to female calves. Moreover, delivering a male calf was more likely to be associated with dystocia (OR: 3.391), with male crossbred calves from Belgian-blue, Angus, and Limousin sires being more frequently involved (OR: 13.687, 2.649, and 5.530, respectively).

The results from the Generalized Linear Model are shown in Table 4 and Table 5. According to the model, parity ≥ 4, calving months of March, April, and from September to November positively influenced the quality of colostrum. Dams carrying a male calf produced a lower quality colostrum compared with those carrying a female calf (−2.78 ± 1.04 Brix degree, *p* = 0.008), while heavier calves were associated with a greater colostrum Brix evaluation (0.29% ± 0.05 for each kg increase, *p* < 0.001). The only factor influencing the serum Brix% of female Holstein calves at two days of life was the day- or night-occurring birth (−0.386 ± 0.188 Brix% in calves born during the night, *p* = 0.04).

## 4. Discussion

The major findings of this work suggest that calving overnight does not affect the Brix% of colostrum. However, calves born overnight and fed the day after had decreased serum Total Protein concentrations as indicated by reduced Brix refractometer readings, compared with calves fed soon after birth.

In this study, 32% of deliveries occurred during the night hours, that is, from 18:00 to 04:59. The prevalence of night-occurring calving was similar to the distribution observed in a previous study [17], while other investigations have either reported an opposite trend, especially in primiparous animals [9], or an equal distribution [18]. Herein, parity was not associated with day- or night-occurring calving. Interestingly, male calves, independently of sire breed, showed a significant association with day-occurring calving. To our knowledge, this is the first report showing a difference in the distribution of calving during the day associated with the sex of the calf. Previous studies [18,19] have provided evidence of the influence of time of feeding on the hour of birth in non-seasonal and seasonally calving cattle. They observed that at-term gestating cattle fed in the evening (approximately from 16:00 to 20:00) calved more frequently the following morning. Gleeson et al. [19] also evaluated the effect of calf sex but found no influence on the hour of calving. In our study, fresh TMR was distributed once a day, and cows were free to move to the feed bunk at any hour; thus, the influence of feeding time on the determination of calving was unlikely in our observation. Concerning the interaction between the sex of the fetus and the hour of birth, an observation on foaling hour in New Zealand standardbred mares showed that female foals were born more frequently during daylight [20]. The solstitial-melatonin-testosterone hypothesis [21] suggests that maternal melatonin secretion, which increases during night hours, also influences the fetal release of testosterone, probably at the adrenal level. Fetal cortisol is essential to the induction of parturition in cattle; as it is also produced by the adrenal gland, we hypothesized that melatonin could influence the hour of birth in our study. However, more investigations are needed to fully explore this mechanism, due to differences in male and female birth distribution found between bovine and equine species.

Calf sex and breed also affected the incidence of calving requiring assistance, with crossbred males from Angus, Limousin, and Belgian-blue sires most frequently involved. The average weight of female Holstein calves at birth is in accordance with that of other investigators [1]. Although the weight of male calves at birth was not recorded in this study, male fetuses are generally associated with longer gestation, which leads to greater live weight at birth and greater calving difficulty [22].

It is generally suggested to collect and feed colostrum soon after calving to avoid both the dilution effect of immune-globulins and the progressive impermeability of the calf’s gut [3,6]. However, in the field context some calvings occur during the night hours, when farm personnel are not present. Unless remote calving alarms are applied, it is likely that those calves will receive colostrum the following morning [23,24,25]. We found no effect on colostrum Brix% when accounting for the hour of calving or the day- or night-calving dichotomous variable. Studies on the effect of collection time on colostrum quality often reported inconclusive results. For example, in the study by Sutter et al. [2], cows that calved during the night (from 22.00 to 6.00) showed decreased colostrum Brix% compared to dams delivering during the day, while Soufleri et al. [6] reported a slight decrease (1 Brix%) in colostrum collected 6 h after parturition. Lessler et al. [26] and Van Keulen et al. [8] found no difference, but in the latter case the authors compared the colostrum of dams milked within 12 h after calving and those milked from 18 to 24 h later and some cows from this group were also suckled. In our study, suckled cows were excluded from the dataset, and the maximum interval from birth to first milking was 11 h in cows that calved during the night. Thus, the difference in study design makes comparison unreliable.

In our observation, the colostrum Brix was 27.43% ± 2.42%, which is greater than that found by Turini et al. [1] but similar to that reported by Sutter et al. [2] and Soufleri et al. [6]. The average reported here was well above the minimum suggested cutoff of 21% [3]. A small percentage of dams had colostrum ≤ 21% Brix or produced less than 4L, thus requiring the thawing of additional or higher quality colostrum. Greater colostrum quality was observed in the months of March, April, and from September to November, as also reported in previous studies [27,28,29]. It is likely that heat stress, especially during the summer months (from June to August), exerted some effect on the quality of colostrum; however, due to the paucity of calving events during the above-mentioned months, the influence of heat stress could not be evaluated in this work. Moreover, without a detailed monthly and/or geographic climate analysis, comparisons show limited scientific soundness [6]. In the study here, the quality of colostrum from primiparous cows was not significantly different from the colostrum collected from second and third parity dams, unlike other reports [1,6]. This finding could be due to the management of pregnant and late-gestating heifers, as they were housed in a dedicated area and were always kept separated from multiparous cows. This likely avoided the social competition for resting, feeding, and drinking space. Moreover, vaccination schemes in the last trimester of pregnancy likely played a role in increasing the Brix% of colostrum. Here, dams carrying a male calf produced lower quality colostrum, different to that reported in other studies [26,30]; moreover, we found that heavier female calves were associated with greater colostrum Brix% evaluation.

Van Keulen et al. [8] demonstrated that a cutoff > 23% for Brix refractometry ensures the transfer of passive immunity even when colostrum is fed 18 h after birth; however, in their observation they found that 20% of calves that received colostrum within 12 h after birth showed FPT. Morin et al. [7] reported 32% of calves with serum Brix < 8.4%. If 8.4% Brix serum cutoff is used, only 4% of the female calves in our study could be classified as at risk of FPT, as previously described by Deelen et al. [31]. The maximum time interval from birth to colostrum administration in calves born during the night in our study was 11 h. The reduced interval from birth to first feeding in calves born during the day, the overall good quality of colostrum, and the standardized quantity fed with an esophageal tube (4 liters) used here likely contributed to ensuring IgG adsorption. A recent investigation on colostrum feeding in Italian dairy herds showed that most of the farms administered only 2 L of good-quality colostrum; this was likely the cause of the great prevalence of FPT reported [32]. According to the results from the Generalized Linear Model, only night birth affected the serum Brix refractometry in female calves in this study. Even though the quality of colostrum fed was not statistically different, calves born during the night and fed the day after had a lower serum Brix% at two days of life. The difference consisted of 0.386% Brix, which is not determinant for FPT.

## 5. Conclusions

Our results suggest that calves born overnight and fed the day after had decreased serum Total Protein concentrations as indicated by reduced Brix refractometer readings, compared with calves born during the day. However, the administration of 4 L of high-quality colostrum proved to be effective in ensuring a serum Brix > 8.4% at two days of life. Where the prevalence of good-quality colostrum is lower, improving calving supervision and ensuring timely feeding are important to reduce the risk of FPT [8]. Calving supervision remains a key element in optimal farm management: quick resolution of dystocia and timely cow–calf separation contribute to calf/dam survival, biosecurity, and the control of diseases characterized by vertical transmission, such as paratuberculosis [32,33,34].

## Figures and Tables

**Table 1:** Distribution of dam parity, dry period length, Brix% of dam colostrum and colostrum fed, serum Brix% of female Holstein calves, and weight at birth.

	N	Mean ± St. Dev.	Minimum	Maximum	Skewness	Kurtosis
Parity	473	2.48 ± 1.51	1	9	0.77	1.20
Dry period length (days)	312	59.73 ± 16.19	29	168	3.93	21.20
Brix colostrum (%)	416	27.43 ± 2.92	16	32	−0.14	−0.21
Brix fed (%)	458	27.56 ± 2.79	20	32	0.02	−0.67
Brix serum (%)	189	10.19 ± 1.10	8	13	10.62	133.50
Weight (kg)	179	37.84 ± 6.01	28.00	55.00	0.80	2.64

N = number of animals included into the database.

**Table 2:** Frequency of calving difficulty.

Calving Difficulty	N	Frequency (%)
Eutocia	413	89.2
Mild dystocia	49	10.58
Severe dystocia	1	0.22
Total	463	100

**Table 3:** Factors associated with day- or night-calving and with the occurrence of dystocia.

Outcome	Variable	Individual Variables	OR (95% CI)	*p*-Value
Calving during the day	Calf sex	Male	1.473 (1.002–2.163)	0.049
		Femal	Referent	
	Sire breed	Angus	1.201 (0.783–1.842)	0.402
		Belgian-blue	0.737 (0.250–2.169)	0.579
		Limousin	1.526 (0.766–3.040)	0.229
		Wagyu	0.875 (0.428–1.788)	0.714
		Holstein	Referent	
	Dam parity		1.010 (0.867–1.177)	0.897
Dystocia	Calf sex	Male	3.391 (1.716–6.700)	<0.001
		Femal	Referent	
	Sire breed	Angus	2.649 (1.235–5.682)	0.012
		Belgian-blue	13.687 (4.091–45.792)	<0.001
		Limousin	5.530 (2.214–13.871)	<0.001
		Wagyu	1.711 (0.458–6.395)	0.425
		Holstein	Referent	
	Dam parity		1.154 (0.933–1.427)	0.188

**Table 4:** Factors affecting colostrum Brix% of dams.

Parameter	Estimate	95% Confidence Interval		
		Lower	Upper	*p*-Value
(Intercept)	9.126	−1.256	19.507	0.085
Parity				
1	Referent			
2	2.012	−510	4.533	0.118
3	2.375	−941	5.691	0.160
≥ 4	3.428	0.544	6.313	0.020
Month				
January	0.480	−1.953	2.913	0.699
February	0.078	−2.731	2.887	0.957
March	4.011	1.478	6.543	0.002
April	5.087	2.316	7.858	<0.001
May	2.376	−1.406	6.157	0.218
June	Not enough cases			
July	−0.102	−3.524	3.320	0.954
August	Not enough cases			
September	3.966	0.277	7.654	0.035
October	5.135	2.051	8.218	0.001
November	6.971	3.772	10.170	<.001
December	Referent			
Calf Sex				
Male	−2.780	−4.826	−735	0.008
Female	Referent			
DayNight				
Day	Referent			
Night	2.080	−0.225	4.385	0.077
Calf death	Not enough cases			
Weight	0.289	0.188	0.390	<0.001
Dry period Length	0.016	−0.103	0.135	0.792
(Scale)	1.400	0.851	2.303	

**Table 5:** Factors affecting serum Brix% of female Holstein calves.

Parameter	Estimate	95% Confidence Interval		
		Lower	Upper	*p*-Value
(Intercept)	9.765	6.480	13.050	<0.001
Brix fed	0.020	−0.045	0.085	0.555
Weight	0.027	−0.027	0.081	0.324
DayNight				
Day	Referent			
Night	−0.386	−0.754	−0.018	0.040
Parity				
1	Referent			
2	−1.041	−2.561	0.479	0.180
3	−0.770	−2.433	0.894	0.364
4	0.116	−1.761	1.992	0.904
5	0			
(Scale)	1.073	0.840	1.371	

## Data Availability

The data presented in this study are available on request from the corresponding author.
